# Dr. Charles Pinto

**Published:** 2009-10

**Authors:** H. S. Adenwalla

**Affiliations:** Head of the Charles Pinto Centre for Cleft Lip and Palate, Jubilee Mission Medical College and Research Institute, Trichur-680 005, India. E-mail: charlespinto@sify.com

**Figure:**
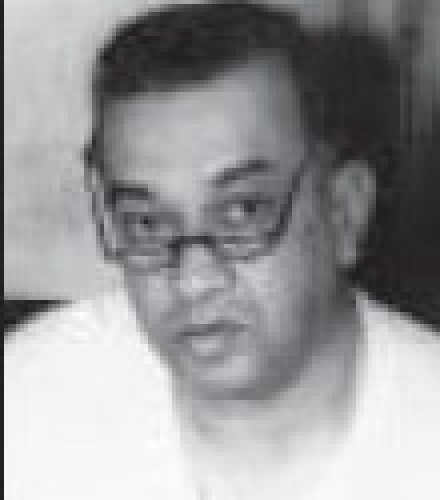
Charles Pinto

When one is asked to write about Charles Pinto, one wonders as to what aspect of this man's character and accomplishments one should concentrate on, for Charles Pinto was indeed a man of many parts and a man for all seasons. One recalls Charles Pinto as the versatile surgeon who gravitated in a short life-span from general to neuro and then to paediatric surgery and ultimately found his haven in the then burgeoning art of plastic surgery. Or should one write about Charles Pinto the teacher whose bed-side clinics were full of the anecdotes about the great masters of yesterday by which he made his subject so alive? I remember Charles Pinto the connoisseur of art and music, of good food and vintage wine; and Charles Pinto an old world knight in armour, chivalrous to the core, a defender of the weak and a champion of lost causes; who would put his own interests second to those of a young resident who was in trouble. In the intimacy of his personal friendships, he had a quality, which was almost feminine in its caressing charm; I have never known him in all his life to fail a friend, shirk a responsibility, or refuse to answer a challenge.

If I am asked as to why this man was so important for the evolution of plastic surgery in this country, I would say that on that well-nigh empty stage, he strode like a colossus with a brave vision for the future of plastic surgery. His enthusiasm for the cause was so infectious that he fired the imagination of young men around him and drove them to superhuman heights of endeavour. He brought to plastic surgery a versatility which was common among the surgeons of those days. He could open and operate in the three body cavities, the skull, the thorax or the abdomen with equal skill. As a teacher of operative surgery it would be hard to find an equal, although a master of the spoken word, he could convey the importance of a certain method of dissection by superb dexterity using both hands. To watch him reconstruct a cleft lip or a palate, or shape a nose or reduce a breast was sheer joy. The ease and speed with which he worked was phenomenal. The show-man in him only came out when he left visitors gaping at the theatre clock. And then over a cup of tea in the side room of the theatre, he rose to scintillating heights; collecting all the young men around him, he would talk on any subject that interested him at the moment. His interests were so wide, his vision panoramic, one almost resented the anaesthetist peeping in and saying “your next case is ready Sir.” It was these sessions that Charles Pinto the Man, more than Charles Pinto the surgeon who inspired future generations of plastic surgeons in this country. This is why the memory of this great teacher lives on.

